# Daily steps are a predictor of, but perhaps not a risk factor for Parkinson’s disease: findings from the UK Biobank

**DOI:** 10.1038/s41531-025-01214-6

**Published:** 2025-11-24

**Authors:** Aidan Acquah, Andrew Creagh, Valentin Hamy, Alaina Shreves, Charilaos Zisou, Charlie Harper, Stefan van Duijvenboden, Chrystalina Antoniades, Derrick Bennett, David Clifton, Aiden Doherty

**Affiliations:** 1 Nuffield Department of Population Health, University of Oxford, Oxford, UK; 2 Big Data Institute, Li Ka Shing Centre for Health Information and Discovery, University of Oxford, Oxford, UK; 3 Institute of Biomedical Engineering, Department of Engineering Science, University of Oxford, Oxford, UK; 4 GSK, London, UK; 5 Division of Cancer Epidemiology and Genetics, National Cancer Institute, National Institutes of Health, Bethesda, MD USA; 6 Nuffield Department of Clinical Neurosciences, Medical Sciences Division, University of Oxford, Oxford, UK; 7 Oxford Suzhou Centre for Advanced Research, University of Oxford, Suzhou, Jiangsu China

**Keywords:** Diseases, Health care, Medical research, Neurology, Neuroscience, Risk factors

## Abstract

Previous studies link lower physical activity with incident Parkinson’s disease (PD) but rely on self-reported data and fail to address reverse causation. This study used accelerometer-derived daily step count, an objective measure of physical activity, to examine its association with incident PD in the UK Biobank, within successive periods of follow-up. PD cases were identified through hospital inpatient and death data, and associated with machine learning-derived step counts using Cox regression models, adjusted for age, sex, demographic and lifestyle factors. For 94,696 valid participants and a median follow-up of 7.9 years, 407 incident PD cases occurred. Every 1000 steps higher were associated with 8% lower risk of PD (hazard ratio 0.92, 95% confidence interval 0.89–0.94). However, this association was no longer statistically significant when excluding follow-up periods closer to the time of accelerometer wear, suggesting that low activity may be an early marker, but not a risk factor for PD.

## Introduction

Parkinson’s disease (PD) is the second most prevalent and most rapidly growing neurodegenerative disorder^1,2^, with an estimated prevalence of 9.4 million^3^ cases in 2020, marking a substantial increase from 5.2 million cases in 2004^4^. The prodromal phase of PD has many well-recognised motor-related symptoms, such as subtle motor dysfunction, that are believed to occur over a decade before diagnosis^5^. Recognising these early indicators could provide crucial insights for improved disease understanding and may offer the possibility of identifying modifiable risk factors for developing PD.

In existing literature, low self-reported physical activity has been associated with a higher risk of incident PD^6,7^. Despite these observations, uncertainties remain, stemming from a reliance on self-report measurements of physical activity, which are crude and less reliable, creating greater uncertainties in observed associations^8,9^. In addition, PD’s progressive and prolonged development poses challenges in distinguishing between incident and prevalent cases. This ambiguity can introduce reverse-causation bias, where the observed associations may be influenced by the change in behaviour of individuals who already have underlying PD, rather than vice-versa. One common approach to mitigate potential reverse causation is to exclude the first few years of follow-up data. In PD studies, there is inconsistency around the number of years removed across studies, ranging between four to ten years, however, reduced activity levels were still significantly associated with incident PD after this exclusion^6^.

Daily step counts serve as a valuable proxy for measuring physical activity levels. One of the primary advantages of using average daily steps over other physical activity metrics is their simplicity, allowing for clear communication and understanding among the general population. This ease of interpretation helps in translating research findings into effective public health messaging^10^. Existing research into the association between physical activity and incident PD have typically used metabolic equivalent of task (MET) scores to estimate physical activity^6^. However, the increasing adoption of wearable devices, capable of estimating step counts, has encouraged research on their accuracy and reliability, particularly in populations diagnosed with PD^11,12^. The translatable nature of daily step counts encourages the expansion of this research into incident PD, allowing for results to offer insight into public health recommendations for potential disease prevention.

We, therefore, aimed to investigate the association between accelerometer-measured daily step counts and incident Parkinson’s disease, and how this association changed with latter windows of follow-up.

## Results

### Participant characteristics

**Fig. 1:**
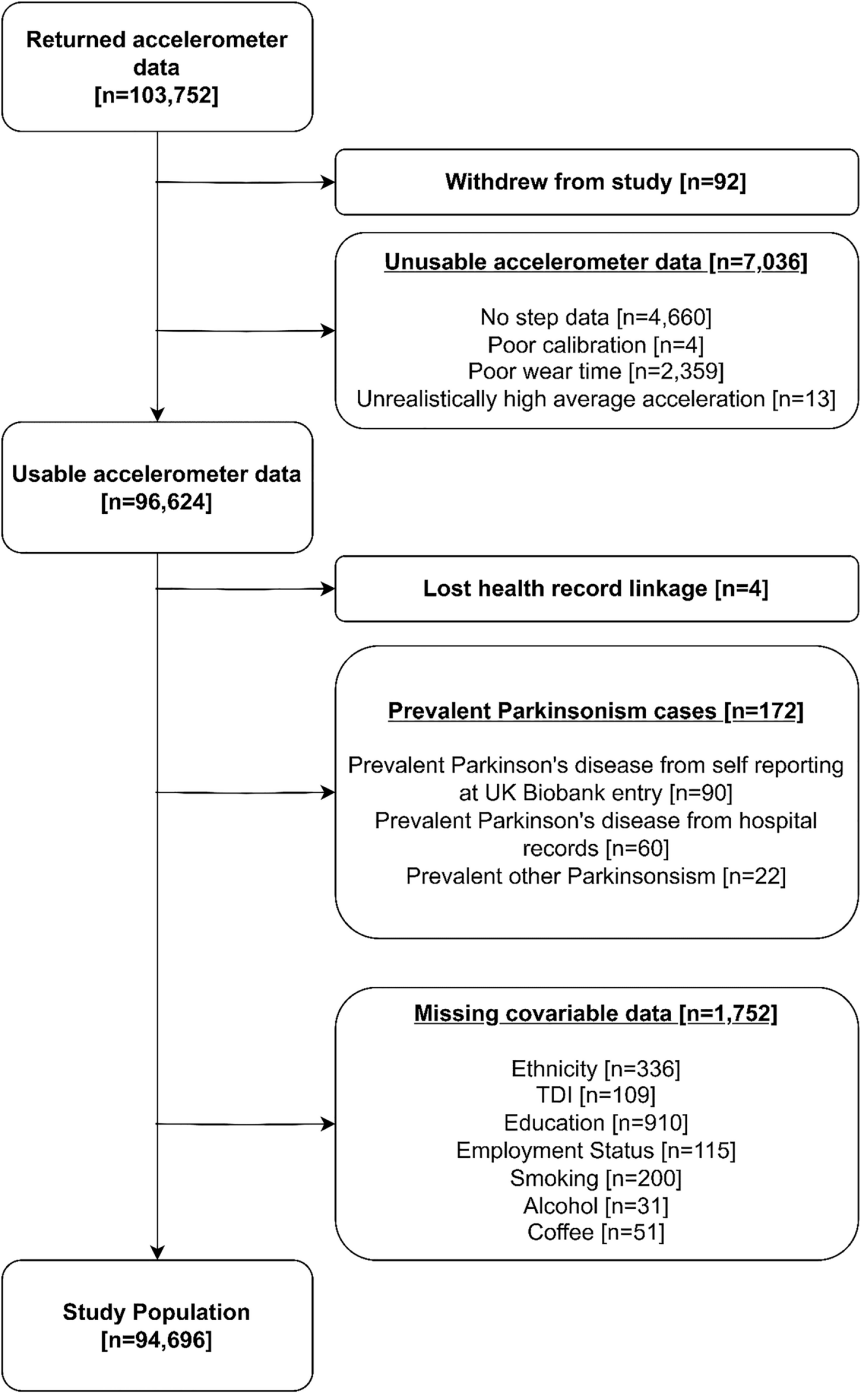
Exclusion flow diagram for analysis of UK Biobank physical activity monitoring population. TDI Townsend deprivation index.

After exclusions, this study consisted of 94,696 UK Biobank physical activity monitoring study^13^ participants (Fig. 1).

As shown in Table 1, among these participants, 56% were female, 97% identified as White. Most were in the least to second least deprived TDI quintile (<−1.509), compared to the TDI distribution of the general UK population in 2011^14^. Those with step counts in the highest quintile (12,369+ daily steps) were younger, and had lower BMI compared to those in the lowest step count group (<6276 daily steps).

**Table 1:** Count by demographic characteristics in the UK Biobank

Characteristic	Median daily step count					All
	<6276	6276–8145	8146–9966	9967–12,368	12,369+	
No. of participants	18,933	18,939	18,941	18,942	18,941	94,696
Demographic factors						
Age, mean (SD), (years)	63.3 (7.96)	62.6 (7.89)	62.3 (7.85)	62.1 (7.74)	61.4 (7.65)	62.3 (7.84)
Female, No. (%)	10,792 (57.0%)	10,916 (57.6%)	10,780 (56.9%)	10,727 (56.6%)	10,158 (53.6%)	53,373 (56.4%)
White ethnicity, No. (%)	18,288 (96.6%)	18,321 (96.7%)	18,377 (97.0%)	18,416 (97.2%)	18,406 (97.2%)	91,808 (97.0%)
Socio-economic factors, no. (%)						
Most deprived UK population TDI Quintile (≥2.516)^14^	2242 (11.8%)	1711 (9.0%)	1720 (9.1%)	1759 (9.3%)	1881 (9.9%)	9313 (9.8%)
No education/professional qualifications	2157 (11.4%)	1643 (8.7%)	1489 (7.9%)	1305 (6.9%)	1270 (6.7%)	7864 (8.3%)
Not employed	8418 (44.5%)	7271 (38.4%)	7133 (37.7%)	6793 (35.9%)	6498 (34.3%)	36,113 (38.1%)
Recruitment centre						
England	16,979 (89.7%)	16,997 (89.7%)	17,079 (90.2%)	16,961 (89.5%)	16,950 (89.5%)	84,966 (89.7%)
Scotland	1147 (6.1%)	1220 (6.4%)	1170 (6.2%)	1270 (6.7%)	1357 (7.2%)	6164 (6.5%)
Wales	807 (4.3%)	722 (3.8%)	692 (3.7%)	711 (3.8%)	634 (3.3%)	3566 (3.8%)
Behavioural factors						
Alcohol 3+ times/week, No. (%)	7732 (40.8%)	8918 (47.1%)	9506 (50.2%)	9988 (52.7%)	10,234 (54.0%)	46,378 (49.0%)
Never smoker, No. (%)	9971 (52.7%)	10,827 (57.2%)	11,025 (58.2%)	11,214 (59.2%)	11,057 (58.4%)	54,094 (57.1%)
Non coffee drinker, No. (%)	4168 (22.0%)	3751 (19.8%)	3611 (19.1%)	3555 (18.8%)	3516 (18.6%)	18,601 (19.6%)
Accelerometer-derived Daily Sleep Duration, mean (SD), (hours)	9.28 (1.53)	8.97 (1.22)	8.82 (1.13)	8.69 (1.09)	8.50 (1.04)	8.85 (1.24)
BMI, mean (SD), (kgm^−2^)	28.6 (5.58)	27.1 (4.45)	26.5 (4.16)	25.9 (3.88)	25.5 (3.68)	26.7 (4.53)
Prior disease, no. (%)						
Type 2 Diabetes	1533 (8.1%)	857 (4.5%)	611 (3.2%)	535 (2.8%)	474 (2.5%)	4010 (4.2%)
Depression	7376 (39.0%)	6534 (34.5%)	6217 (32.8%)	5928 (31.3%)	5487 (29.0%)	31,542 (33.3%)
Constipation	1020 (5.4%)	707 (3.7%)	652 (3.4%)	627 (3.3%)	548 (2.9%)	3554 (3.8%)
Bladder dysfunction	177 (0.9%)	122 (0.6%)	115 (0.6%)	89 (0.5%)	80 (0.4%)	583 (0.6%)
Season of device wear, no. (%)						
Spring	4113 (21.7%)	4160 (22.0%)	4432 (23.4%)	4413 (23.3%)	4604 (24.3%)	21,722 (22.9%)
Summer	4334 (22.9%)	4536 (24.0%)	4989 (26.3%)	5378 (28.4%)	5897 (31.1%)	25,134 (26.5%)
Autumn	5675 (30.0%)	5830 (30.8%)	5663 (29.9%)	5459 (28.8%)	5305 (28.0%)	27,932 (29.5%)
Winter	4811 (25.4%)	4413 (23.3%)	3857 (20.4%)	3692 (19.5%)	3135 (16.6%)	19,908 (21.0%)

*TDI* Townsend deprivation index, *BMI* Body Mass Index, *SD* standard deviation.

The overall mean of median daily steps calculated in the study population was 9446 steps and the standard deviation was 3822 steps. Participants with prevalent PD (*n* = 150), who were excluded from the study, exhibited the lowest average step count, as compared to incident and non-PD participants. Among those with incident PD, average median daily step counts were progressively higher with longer follow-up time from accelerometer wear to the first reporting of PD in their health records. Despite this, the average median daily steps in the incident PD population remained lower than that of non-PD participants, as illustrated in Supplementary Fig. 2.

### Association of daily steps with incident Parkinson’s disease

**Fig. 2:**
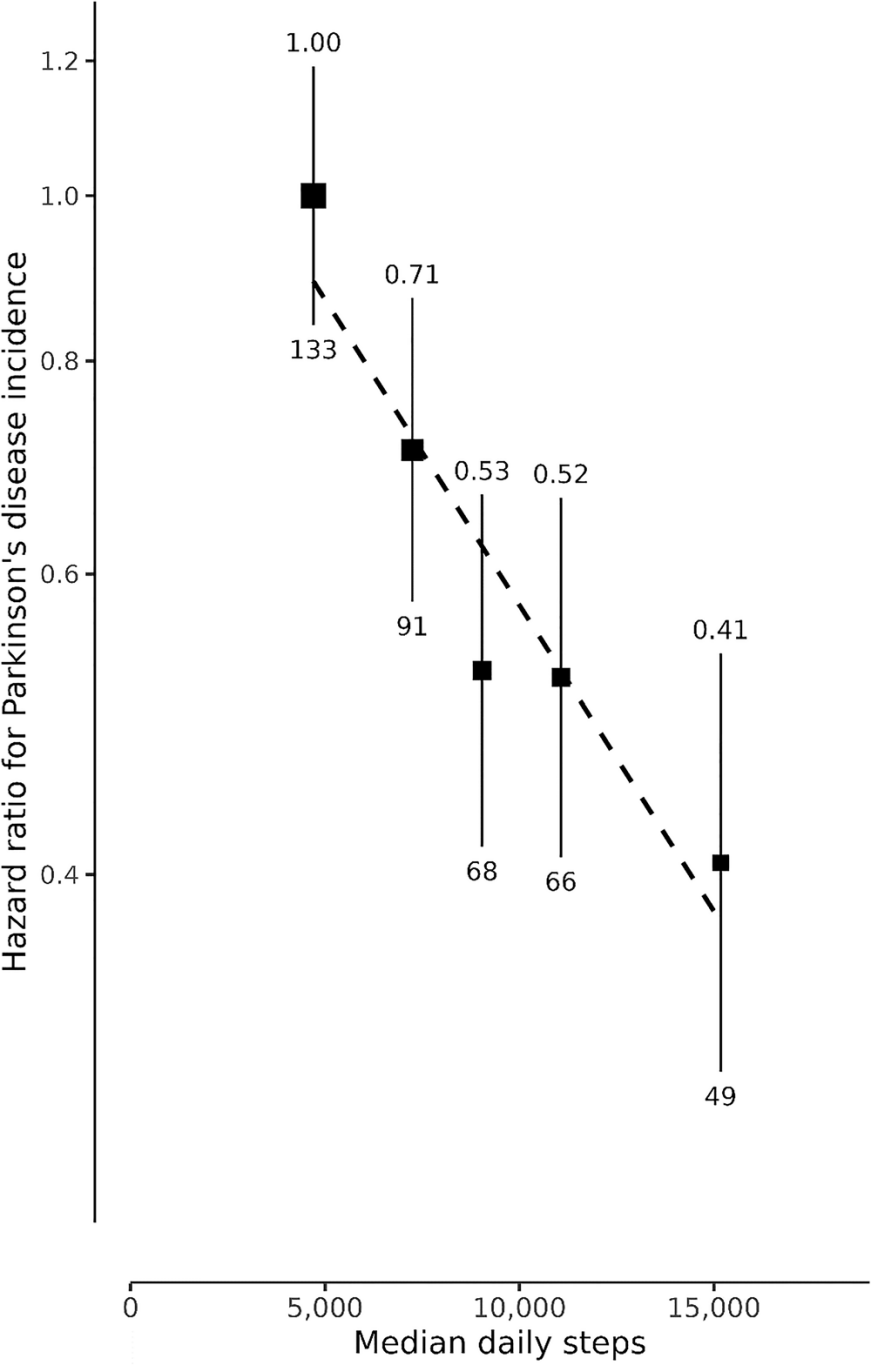
Association between quintiles of median daily step count and incident Parkinson’s disease. Hazard ratios (HR) and 95% group-specific confidence intervals (CI) were calculated using age as a timescale, adjusted for season of wear, sex, ethnicity, Townsend deprivation index, geographic region, education, employment, alcohol intake, smoking status and coffee intake. HR is above, and the number of events is plotted below each data point. The HR (95% CI) per 1000 additional daily steps is 0.92 (0.89–0.94).

Participants had a median follow-up time of 7.9 years (IQR: 7.4, 8.4), during which 407 incident PD cases occurred, with a median time to diagnosis of 5.2 years (IQR: 3.1, 6.8). We observed a strong and significant inverse linear association between median daily step quintiles and incident PD using data from the entire duration of follow-up (Fig. 2). Those who walked over 12,369 steps daily had a 59% lower risk of PD (HR 0.41; group specific 95% CI: 0.31–0.54), compared to those walking less than 6276 steps daily (HR 1.00; group-specific 95% CI: 0.84–1.19), after multivariable adjustment. There was no evidence of departure from linearity (*p* = 0.65).

For the multivariable model, every 1000 daily steps higher was associated with an 8% lower risk of PD (HR 0.92; 95% CI 0.89–0.94). The sequential adjustment of covariates from the univariable to multivariable model showed minimal changes to the strength of the association and the chi-squared increased by 33% (shown in the supplementary material). The further adjustment of additional covariates minimally altered the observed associations. However, adjustment for sleep duration did attenuate the strength of association, with a reduction in the chi-squared by 64%, as shown in Supplementary Fig. 5. Furthermore, we observed no significant difference across subgroups, including age groups, sex, BMI status and history of depression. After removing 9734 individuals with prevalent neurological cases, the observed association was similar to that observed in our main study sample (HR 0.92; 95% CI 0.89–0.95).

**Fig. 3:**
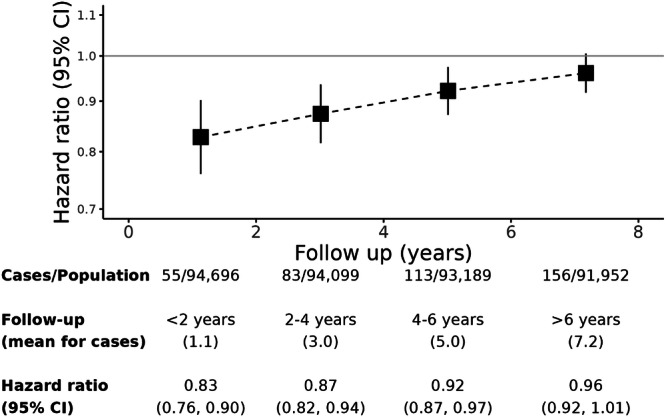
Hazard ratio for incident Parkinson’s disease by period of follow-up per 1000 median daily steps higher. Models use age as timescale, and are adjusted for season of wear, sex, Townsend deprivation index, geographic region of assessment centre, educational attainment, employment, smoking, alcohol and coffee consumption. CI confidence interval.

To address potential reverse causality, we assessed how the association between step counts and PD risk may have changed over follow-up periods of <2, 2–4, 4–6 and 6+ (Fig. 3). We observed the strongest association within the first two years of follow-up (HR 0.83; 95% CI 0.70–0.90), during which there were 55 incident cases (Fig. 3). However, the association was attenuated for later windows of follow-up, trending towards not statistically significant. This is despite having higher relative numbers of cases in latter periods of follow-up, reducing uncertainty in estimates. For the follow-up period of over 6 years, the HR was no longer statistically significant, with a hazard of 0.96 (95% CI: 0.92–1.01).

These findings remained consistent after running a sensitivity analysis using a step count algorithm trained on both healthy and PD populations. The estimate median daily step counts between the two algorithms, healthy and PD-trained compared to healthy-only trained, showed very strong correlation, with a Pearson correlation coefficient^15^ of 0.97, as seen in Supplementary Fig. 6. Furthermore, a significant association was found between PD-trained median daily step count and incident PD using all periods of follow-up, with covariate adjustment, as shown in Supplementary Figs. 7 and 8. As seen previously, this HR, however, was no longer statically significant when considering follow-up periods of over 6 years (HR 0.96; 95% CI 0.92–1.00).

## Discussion

Higher accelerometer-derived physical activity levels, indicated by median daily step counts, were associated with a lower risk of incident Parkinson’s disease (PD) when using data from all periods of follow-up. This association remained statistically significant after multivariable adjustment, investigating potential confounding factors, and among subpopulations of interest. However, this association was no longer statically significant when considering later periods of follow-up, indicating that reverse causation bias is likely.

Our findings are in contrast to some previous literature in the field that supports an association between physical activity levels and incident PD, even after the consideration of reverse causation^6,16–18^. In a meta-analysis of this association with a median (range) follow-up of 12 (6.1–22.0) years, the relative risk for incident PD comparing the highest and lowest categories of total physical activity was 0.79 (95% CI 0.68–0.91), and remained significant when the first four to ten years of follow-up was removed from the constituent studies (0.76; 95% CI 0.64–0.91)^6^. In our study, removing years of follow-up reduced the strength of the observed association. Similar attenuations between physical activity and diseases of ageing have been reported in other studies. Our findings are more consistent with results from a study on physical activity and dementia that was conducted in the Whitehall Study and Million Women Study^19,20^.

These results, however, support existing literature in finding lower physical activity levels to be a strong indicator/feature of prodromal or early PD^18,21^. One study reported that this reduction in activity may occur up to 4 years before a clinical diagnosis^18^. A separate study assessing minutes of daily physical activity^21^ found a reduction of 29% in comparing clinical PD participants to controls, in minutes of daily physical activity. There are several hypotheses for these activity changes, including changes in overall disability, poorer walking performance, and fear of falls/anxiety/depression^21^. It should be noted, however, that this reduction in activity is not fully explained by known factors, with this study encouraging further research into explaining this behaviour^21^.

Our study has several strengths. First, we used objective measures of activity derived from accelerometer data, which offers greater reliability in the measurement of this exposure compared to questionnaire-based studies. Second, the use of step count as a measure of daily physical activity offers the opportunity for better communication as step counts are easily translatable for public health messaging. Next, this work has built on previous studies investigating PD using the UK Biobank physical activity monitoring study^16,17,22^, however with longer follow-up, while also addressing some of these studies’ limitations. Of these limitations, we explored how the association may change over follow-up periods to better describe the potential impact of reverse causation.

There are also some limitations to our study. Firstly, this analysis was purely observational; hence we cannot exclude the possibility of unmeasured or residual confounding in the observed associations, despite considering a comprehensive set of confounders. Secondly, the UK Biobank provides limited health data linkage for participants, which we use to identify participants with PD. We use the approach recommended by UK Biobank to ascertain PD, combining hospital inpatient, death register and self-reported data^23^, which may tend to identify more severe cases. These records do not provide information regarding the severity or subtype of PD, which may offer further insights into this association. Furthermore, primary care data could bolster PD cases, but is currently limited to just 45% of participants and only up to 2017^13^. Lastly, this study is limited by the number of cases observed within period of follow-up. With 407 incident cases of PD from all periods of follow-up, and just 156 diagnosed at least 6 years after participating in the study, we are somewhat limited in power to observe small effects of greater activity. We are also limited by a maximum follow-up time of 9.4 years. The PD prodromal window is typically reported to be at least 10 years prior to a clinical diagnosis^5^.

Future studies in this population can benefit from higher case numbers from a longer follow-up period, while also giving further insights into this association, beyond the prodromal window of PD. Further investigations should also explore the association between other accelerometer-derived metrics and incident PD, such as metrics relating to gait speed, stride length or the distribution of steps throughout the week. Furthermore, we would encourage similar analyses in other study populations, such as the Parkinson’s Progression Markers Initiative (PPMI)-Verily study^24,25^, containing a higher proportion of individuals with prodromal PD, and the All of Us study^26^, containing greater diversity in race, ethnicity and socio-economic status.

Results from this prospective study add to the literature on physical activity and incident PD, suggesting that higher daily step counts were associated with lower risk of incident PD, but only for short follow-up periods. Therefore, the observed association may be explained by reverse causation bias. This work supports the continued labelling of low physical activity as a marker for Parkinson’s disease, rather than as a risk factor leading to Parkinson’s disease.

## Methods

### Study cohort

The UK Biobank is a prospective cohort study of 502,536 adults in the United Kingdom who were recruited between 2006 and 2010. The consented participants completed a questionnaire, interview, and provided physical measurements, as well as blood, urine, and saliva samples^27,28^. A subgroup of participants consented to wear an Axivity AX3 wrist-worn accelerometer for up to 7 days between 2013 and 2015^13^. All participants provided written informed consent and the study was approved by the National Information Governance Board for Health and Social Care and the National Health Service North West Multicentre Research Ethics Committee (06/MRE08/65).

### Accelerometer-derived physical activity processing

Accelerometer data was processed to derive step counts using the OxWearables “stepcount” package (version 2.1.5), a hybrid self-supervised machine learning model that was trained on ground truth free-living data and counts peaks in detected walking windows of behaviour^29^. Steps were predicted for each participant’s full period of collected data, with missing periods due to non-wear imputed by averaging the step counts in the corresponding times across valid days^29^. We then calculated the median of the imputed daily step counts, across the period of monitoring, as the primary exposure in this study.

### Ascertainment of Parkinson’s disease

The diagnosis of PD was obtained from a combination of the UK Biobank’s linked hospital data; inpatient hospital admissions data, and death data. Diagnoses were coded according to the International Classification of Diseases (version 10) coding system, with outcomes recorded as the first instance of a G20 diagnostic code^23^. This follows the approach recommended by the UK Biobank^23^, and used by other studies in studying PD in the physical activity monitoring cohort^16,17,22^. Incident PD however was defined at their first diagnosis of PD. Participants were censored at the first date of PD diagnosis, date of death, or at the end of their follow-up period (31 October 2022 for England, 31 August 2022 for Scotland, and 31 May 2022 for Wales), whichever came first^30^.

### Statistical analysis

We processed the accelerometer data from 103,660 participants. We excluded participants with device calibration or data reading errors (>1% of values outside +/-8g range), and those whose data could not be processed. In addition, participants were excluded due to inadequate wear time, either less than 72 h of detected wear, or non-wear at a particular time of day for all days of monitoring. We did not implement any day level exclusions, to remove any data due to insufficient wear on a given day. Further exclusions were made for unreasonably high average acceleration (>100 mg).This exclusion criteria aligns with numerous existing studies on this dataset^22,29,31,32^. We also excluded all 150 prevalent PD and 22 other Parkinsonism cases, diagnosed before accelerometer wear, and participants with missing healthcare linkages or covariate data. The final analysis included 94,696 participants (Fig. 1).

A Cox proportional hazard regression model^33^ was used to estimate adjusted hazard ratios (HRs) with 95% confidence intervals (CIs) for the association between daily step counts and incident PD. The association was first evaluated using quintiles of median daily step count. CIs when using quintiles of daily steps as the primary exposure were estimated using floating absolute risk, to represent group-specific variances in hazard, therefore referred to as group-specific CIs^34^.

We then evaluated how the risk of PD was associated with each 1000 steps per day higher. We sequentially applied the full set of adjustments to the multivariable model. The impact of these sequential adjustments were assessed using chi-squared test. Specifically, for the per-1000 median daily steps with one degree of freedom, we compared the chi-squared values before and after adjustment. A likelihood ratio test comparing quintiles of daily steps as categorical and ordinal was performed to test that the association between daily steps and incident PD did not significantly depart from linearity^35^. This allows for the reporting of hazard ratios per 1000 steps rather than quintiles of step count. At each step of this sequential adjustment, confounding was assessed by observing the chi-squared from a likelihood ratio test of the association.

Multivariable models used age as the time scale, as age was a strong potential confounder in this analysis^36,37^. These models were adjusted for the following covariates: season of device wear (spring, summer, autumn, winter), sex (female, male), ethnicity (white, non-white), Townsend Deprivation Index (TDI, stratified by quintiles in UK population^14^), geographic region of assessment centre (London, Midlands, Yorkshire, Northeast, Northwest, Southeast, Southwest England, Scotland, Wales), educational attainment (degree, diploma, A/AS levels, professional qualification, GCSE/O levels, no degree/qualification), employment status (employed, not employed), smoking status (never, previous, current) and alcohol consumption (never, <3 times per week, ≥3 times per week) and coffee intake (non-drinker, ≤2 cups a day, >2 cups a day). We adjusted for season of device wear as a technical covariate, which explains part of the variation in physical activity levels^38^. The remaining covariates were chosen as known risk factors for PD, particularly those from the PREDICT-PD risk model^36^, with possible association to physical activity, and reliable and accurate recording in UK Biobank records, while not hypothesised to be on the causal pathway^36,39^. All covariate data were provided by participants at the UK Biobank baseline assessment visit, with details provided in Supplementary Table 2. The proportional hazards assumption was tested using Schoenfeld residuals^40^, and no violations were observed.

We conducted several orthogonal analyses using the models assessing the risk for each 1000 daily steps higher. First, we expanded our multivariable-adjusted models by additionally adjusting for body mass index (BMI) at baseline, depression, type 2 diabetes, constipation, bladder dysfunction derived through a combination of self-report and health records, and sleep duration derived from accelerometer data. These covariates were chosen as known health-related PD risk factors with an association with physical activity, that were hypothesised possibly be on the causal pathway^5,36,41^. We then explored whether the observed associations differed within subpopulations of interest, including by age groups, sex, BMI (<25, 25–30, 30+ kg/m^2^)^42^ and history of depression. Additionally, we performed a sensitivity analysis examining the associations after excluding participants with any history of neurological disorder. To assess potential reverse causation and evaluate how associations changed over time, we estimated HRs within successive follow-up intervals (0–2, 2–4, 4–6, and ≥6 years) using Lexis expansion^43,44^. For each participant, total follow-up time was divided into these intervals, generating one record for each period in which the individual contributed person-time. Each record represented time at risk within that interval, with follow-up left-truncated at the start and right-censored at the end of the interval. HRs were then estimated separately for each interval to assess temporal changes in the associations, following a similar approach to that described by Floud et al.^20^.

We also performed a final sensitivity analysis, to ensure the model could reliably detect steps in individuals at-risk of incident PD. To do so, we modified the stepcount algorithm deployed on the UK Biobank population, so that instead of only being trained on the healthy OxWalk population, it was additionally trained on the Michael J. Fox Foundation Levodopa Response (MJFF-LR) dataset^45^, consisting of wrist-worn accelerometer data collected from 28 clinical PD individuals. The full association analysis pipeline was then re-run, using this PD-trained stepcount algorithm, to observe how this may affect our findings.

Statistical analyses were performed using R, version 4.3.2, using Cox regression models from the survival package, version 3.8-3. Results were reported according to Strengthening the Reporting of Observational Studies in Epidemiology (STROBE) guidelines (Supplementary Table 3)^46^.

## Supplementary information

Supplementary information

## Data Availability

This study uses UK Biobank data, available at: [https://www.ukbiobank.ac.uk/enable-your-research/apply-for-access].

## Code availability

The underlying code for this study is available in Github and can be accessed via this link https://github.com/OxWearables/steps_PD.
