# Supplementary material for: Daily steps are a predictor of, but perhaps not a risk factor for Parkinson’s disease: findings from the UK Biobank
Source: NPJ Parkinsons Dis. 2025 Nov 24;11:355. doi: 10.1038/s41531-025-01214-6 (PMC12722246; doi:10.1038/s41531-025-01214-6)
Supplement: Supplementary file 1 — Supplementary information [file 41531_2025_1214_MOESM1_ESM.docx]

**Supplementary materials**

# **Tables**

Supplementary Table 1: Description of datasets used in this analysis.

| Characteristic | OxWalk study | Michael J. Fox Foundation Levodopa Response study | UK Biobank Physical Activity Cohort |
| --- | --- | --- | --- |
| No. of participants | 39 | 28 | 103,660 |
| Sensor | Axivity AX3 | GENEActiv | Axivity AX3 |
| Raw sampling rate | 100Hz | 50Hz | 100Hz |
| Body location | Dominant wrist | Wrist of most affected limb | Dominant wrist |
| Activity protocol | Unscripted free-living | Scripted tasks | Unscripted free-living |
| Measurement window, per participant | ~1 hour | ~2 hours | Up to 7 days |
| Ground truth capture | Waist worn, foot facing, video camera | Task diaries / Inspection | None |
| Participant Age  Mean (Standard deviation) | 38.5 (14.0)  Range: 19 - 81 | 62.5 (8.8)  Range: 46 - 80 | 62.4 (7.8)  Range: 43 - 79 |
| Participant Sex | 19 Female, 20 Male | 9 Female, 19 Male | 58,267 Female,  45,393 Male |

Supplementary Table 2: Definition of variables from the UK Biobank data

| Characteristic | Source | Notes | UK Biobank field | Coding Notes |
| --- | --- | --- | --- | --- |
| OUTCOME | | | | |
| Age at first PD event | Hospital episode statistics (HES)  records | First ICD-10 code (G20) in HES data. | Derived from 2000 |  |
| Age loss-to-follow up | Death Registry |  | Derived from 100093 |  |
| EXPOSURE | | | | |
| Median Daily Step count | Accelerometry | Derived using the “stepcount” tool, version 2.1.5.  Details can be found at: https://github.com/OxWearables/stepcount |  | Reported either as divided into quintiles:  <6,276, 6,276-8,145, 8,146-9,966, 9,967-12,368, 12,369+. or as per-1000 daily steps |
| EXCLUSION VARIABLES | |  |  |  |
| Prior Parkinsonism | HES records | PD-related ICD-10 codes in hospital records before accelerometry wear date:  G20-26, G35, G47 | Derived from 2000 |  |
|  | Baseline | PD-related self-reported disease | Derived from 20002 |  |
|  | Algorithmically derived first instance of PD (G20) | Date G20 first reported (Parkinson’s disease) prior to the accelerometry wear date | Derived from 131022 |  |
| ADJUSTMENT VARIABLES | | | | |
| Age | Baseline | Attained age was the underlying timescale in survival analyses; participants entered the study at the end of accelerometer wear. | Derived from 90011, 34, 52 |  |
| Sex | Baseline |  | 31 | Female, Male |
| Ethnicity | Baseline |  | Derived from 21000 | White, non-white |
| Townsend Deprivation Index | Baseline | Townsend Deprivation Index of address at time of UKB baseline assessment. | Derived from 22189 |  |
| UK recruitment centre geographic region | Baseline |  | Derived from 54 | London, Midlands, Yorkshire, northeast, northwest, southeast, southwest England, Scotland, Wales |
| Education/Qualifications | Baseline | Selected as highest educational achievement. Labels are ordered by highest to lowest value. [ISCED](https://ec.europa.eu/eurostat/statistics-explained/index.php?title=International_Standard_Classification_of_Education_(ISCED)#Background)  [UK Biobank Paper](https://www.ncbi.nlm.nih.gov/pmc/articles/PMC9189971/#sup1) | Derived from 6138 | College/University degree, NVQ/HND/HNC or equivalent, Other professional qualification, A levels/AS levels or equivalent, GCSEs/CSEs/O levels or equivalent, No qualification |
| Employment status | Baseline |  | Derived from 6142, 20119 | Employed, Not employed |
| Smoking status | Baseline |  | Derived from 20116 | Never, previous, current |
| Alcohol consumption | Baseline |  | Derived from 1558 | Never, <3 times per week, ≥3 times per week |
| Coffee intake | Baseline |  | Derived from 1498 | Non-drinker, Drinker - up to 2 cups, Drinker - more than 2 cups |
| ADJUSTMENT VARIABLES – SENSITIVITY ANALYSIS | | | | |
| Body mass index | Baseline |  | Derived from 21001 |  |
| Average sleep duration | Accelerometry |  | Derived from 40046 |  |
| Type 2 Diabetes | HES records | ICD-10 code (E11) in HES data prior to end of accelerometer wear. | Derived from 2000 |  |
|  | Baseline | Type 2 Diabetes self-reported disease at baseline | Derived from 20002, 2443 |  |
| Depression | HES records | ICD-10 code (F32, 33) in HES data prior to end of accelerometer wear. | Derived from 2000 |  |
|  | Baseline | Depression self-reported disease at baseline | Derived from 20002, 2090, 2100 |  |
| Constipation | HES records | ICD10 code (K59.0) in HES data prior to accelerometer wear | Derived from 2000 |  |
|  | Baseline | Use of laxatives in self-reported medication use at baseline | Derived from 6154 |  |
| Bladder dysfunction | HES records | ICD10 code (N31) in HES data prior to accelerometer wear | Derived from 2000 |  |
|  | Baseline | Incontinence self-reported disease at baseline | Derived from 20002 |  |
| Neurological conditions | HES records | Any of the following ICD10 codes in HES data prior to accelerometer wear: A80, A80.0, A80.1, A80.2, A80.3, A80.4, A80.8, A80.9, D32, D36, E10.4, E11.4, E12.4, E13.4, E14.4, F00, F00.0, F00.1, F00.2, F00.9, F01, F01.0, F01.1, F01.2, F01.3, F01.8, F01.9, F02, F02.0, F02.1, F02.2, F02.3, F02.4, F02.8, F03, F05, F05.1, G00, G01, G02, G03, G04, G06, G07, G12, G25.0, G30, G30.0, G30.1, G30.8, G30.9, G31, G31.1, G32, G35, G36, G37, G40, G43, G44, G50, G50.0, G50.1, G50.8, G50.9, G51, G51.0, G52, G53, G57, G60, G61, G61.0, G62, G70.0, G72, G80, G81, G82, G82.2, G82.3, G82.4, G83, G83.0, G83.1, G83.2, G83.3, G83.4, G83.8, G83.9, G84, G90, G91, G92, G93, G93.0, G93.1, G93.2, G93.3, G93.4, G93.5, G93.6, G93.7, G93.8, G93.9, G94, G95, G96, G96.0, G96.1, G96.8, G96.9, G97, G98, G99, H53, H81, H81.0, H81.1, H81.2, H81.3, H81.4, H81.8, H81.9, H83, H90, H91, H91.0, H91.1, H91.2, H91.3, H91.8, H91.9, H93, H93.0, H93.1, H93.2, H93.3, H93.8, H93.9, I67, I67.0, I67.1, I67.2, I67.3, I67.4, I67.5, I67.6, I67.7, I67.8, I67.9, K56, M79, M79.0, M79.1, M79.2, M79.3, M79.4, M79.6, M79.7, M79.8, M79.9, N08, N08.0, N08.8, N08.9, N13, N13.0, N13.1, N13.2, N13.3, N13.4, N13.5, N13.6, N13.7, N13.8, N13.9, Q05, R29.0, R41.81, S0[0-9], S14, S14.0, S24, S24.0, S24.1, S34, S34.0,  S34.1, S34.3, S44, S54, S64, S74, S84, S94, T04, T06, T07, T14, T24, T84, T90, T90.3, T90.4, T90.5, T90.8, T90.9, T91, T91.3 | Derived from 2000 |  |
|  | Baseline | Any of the following self-reported diseases at baseline: infection of nervous system, brain abscess/intracranial abscess, encephalitis, meningitis, spinal abscess, cranial nerve problem/palsy, bell's palsy/facial nerve palsy, trigemminal neuralgia, spinal cord disorder, paraplegia, spina bifida, peripheral nerve disorder, peripheral neuropathy, acute infective polyneuritis/guillain-barre syndrome, trapped nerve/compressed nerve, diabetic neuropathy/ulcers, chronic/degenerative neurological problem, motor neurone disease, myasthenia gravis, multiple sclerosis, parkinsons, dementia, alzheimers, cognitive impairment, other demyelinating disease, epilepsy, migraine, cerebral palsy, other neurological problem, headaches, benign / essential tremor, polio / poliomyelitis, meningioma / benign meningeal tumour, benign neuroma, neurological injury/trauma, head injury, spinal injury, peripheral nerve injury | Derived from 20002 |  |
| DESCRIPTIVE VARIABLES – DESCRIPTIVE TABLES | | | | |
| Wear season |  |  | Derived from 90001 | Winter, Spring, Summer, Autumn |

Supplementary Table 3: Strengthening the Reporting of Observational Studies in Epidemiology (STROBE) guidelines checklist for reporting of findings with this work. Pages with the S prefix correspond to supplementary material, otherwise refer to the main manuscript.

| **Section** | **Subsection** | **Summary** | **Code** | **Pages** |
| --- | --- | --- | --- | --- |
| Title and Abstract | Title and Abstract | Indicate study’s design in title/abstract | 1a | 1-3 |
| Title and Abstract | Title and Abstract | Provide informative summary of study | 1b | 2-3 |
| Introduction | Background/rationale | Explain scientific background and rationale | 2 | 4 |
| Introduction | Objectives | State specific objectives and hypotheses | 3 | 4 |
| Methods | Study design | Present key elements of study design | 4 | 4-6 |
| Methods | Setting | Describe setting, locations, and relevant dates | 5 | 4 |
| Methods | Participants | Give eligibility criteria and selection methods | 6a | 4-5 |
| Methods | Participants | Provide matching criteria for matched studies | 6b | N/A |
| Methods | Variables | Define outcomes, exposures, confounders, and effect modifiers | 7 | 5-6 |
| Methods | Data sources measurement | Give sources and methods of data assessment | 8 | 5-6 |
| Methods | Bias | Describe efforts to address bias | 9 | 6 |
| Methods | Study size | Explain how study size was determined | 10 | N/A |
| Methods | Quantitative variables | Explain handling of quantitative variables | 11 | 5 |
| Methods | Statistical methods | Describe all statistical methods used | 12a | 5 |
| Methods | Statistical methods | Describe methods for examining subgroups and interactions | 12b | 6 |
| Methods | Statistical methods | Explain how missing data were addressed | 12c | 5 |
| Methods | Statistical methods | Explain how loss to follow-up was addressed | 12d | 6 |
| Methods | Statistical methods | Describe any sensitivity analyses | 12e | 6 |
| Results | Participants | Report numbers at each stage of the study | 13a | 6 |
| Results | Participants | Give reasons for non-participation | 13b | N/A |
| Results | Participants | Consider use of a flow diagram | 13c | 16 |
| Results | Descriptive data | Provide characteristics of study participants | 14a | 6-7 |
| Results | Descriptive data | Indicate number of participants with missing data | 14b | 16 |
| Results | Descriptive data | Summarise follow-up time | 14c | 6 |
| Results | Outcome data | Report numbers of outcome events or summary measures | 15 | 6 |
| Results | Main results | Give unadjusted and adjusted estimates with precision | 16a | 7-8, 17-18, S11 |
| Results | Main results | Report category boundaries when continuous variables were categorized | 16b | S3-7 |
| Results | Main results | Translate estimates of relative risk into absolute risk if relevant | 16c | N/A |
| Results | Other analyses | Report other analyses done | 17 | 7-8 |
| Discussion | Key results | Summarise key results with reference to objectives | 18 | 8 |
| Discussion | Limitations | Discuss the limitations of the study | 19 | 9 |
| Discussion | Interpretation | Provide overall interpretation of results | 20 | 8-9 |
| Discussion | Generalisability | Discuss the generalisability of the study results | 21 | 9 |
| Other information | Funding | Give the source of funding and role of funders | 22 | 3 |

## **Figures**


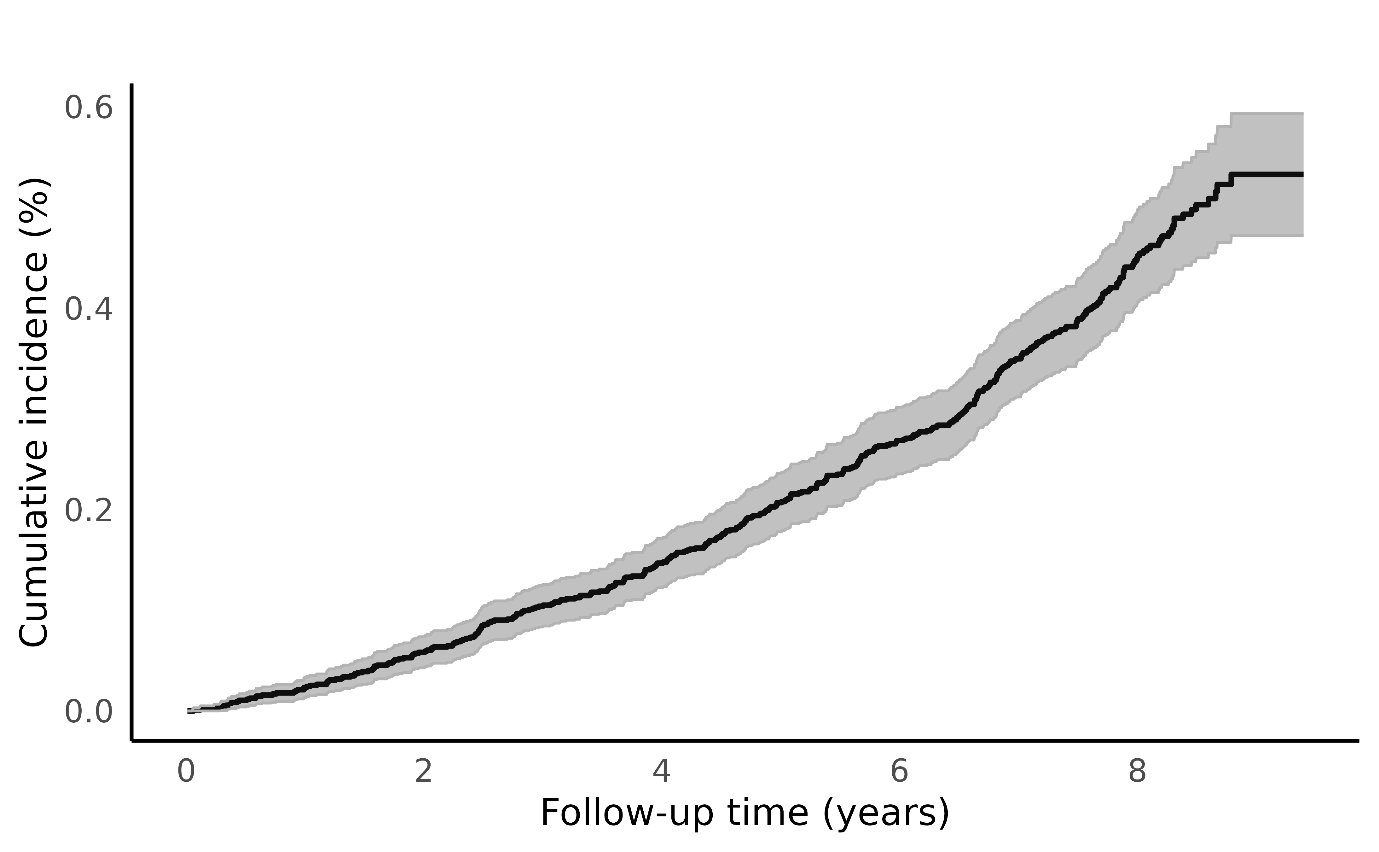


Supplementary Figure 1: Kaplan-Meier curve for cumulative incidence of Parkinson's disease in the UK Biobank physical activity monitoring study population.


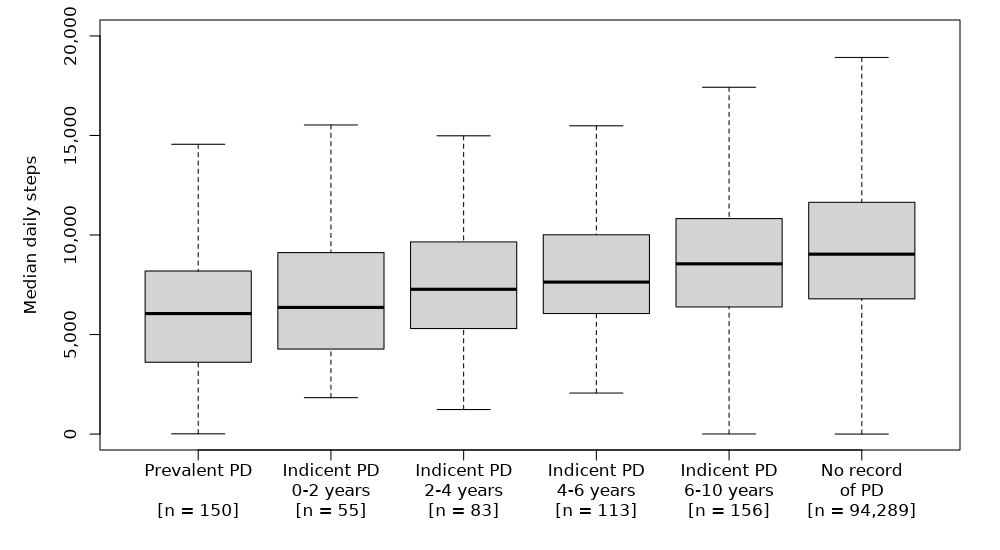


*Supplementary Figure* 2*: Box plots for median daily steps over seven days taken by participants of the UK Biobank physical activity monitoring study, excluding outliers. Incident Parkinson’s disease cases are separated by the interval of follow-up, from the time between accelerometer device wear and the first reporting of Parkinson’s disease in their health records. The population for each grouping of Parkinson’s disease status is shown by n in square brackets.*
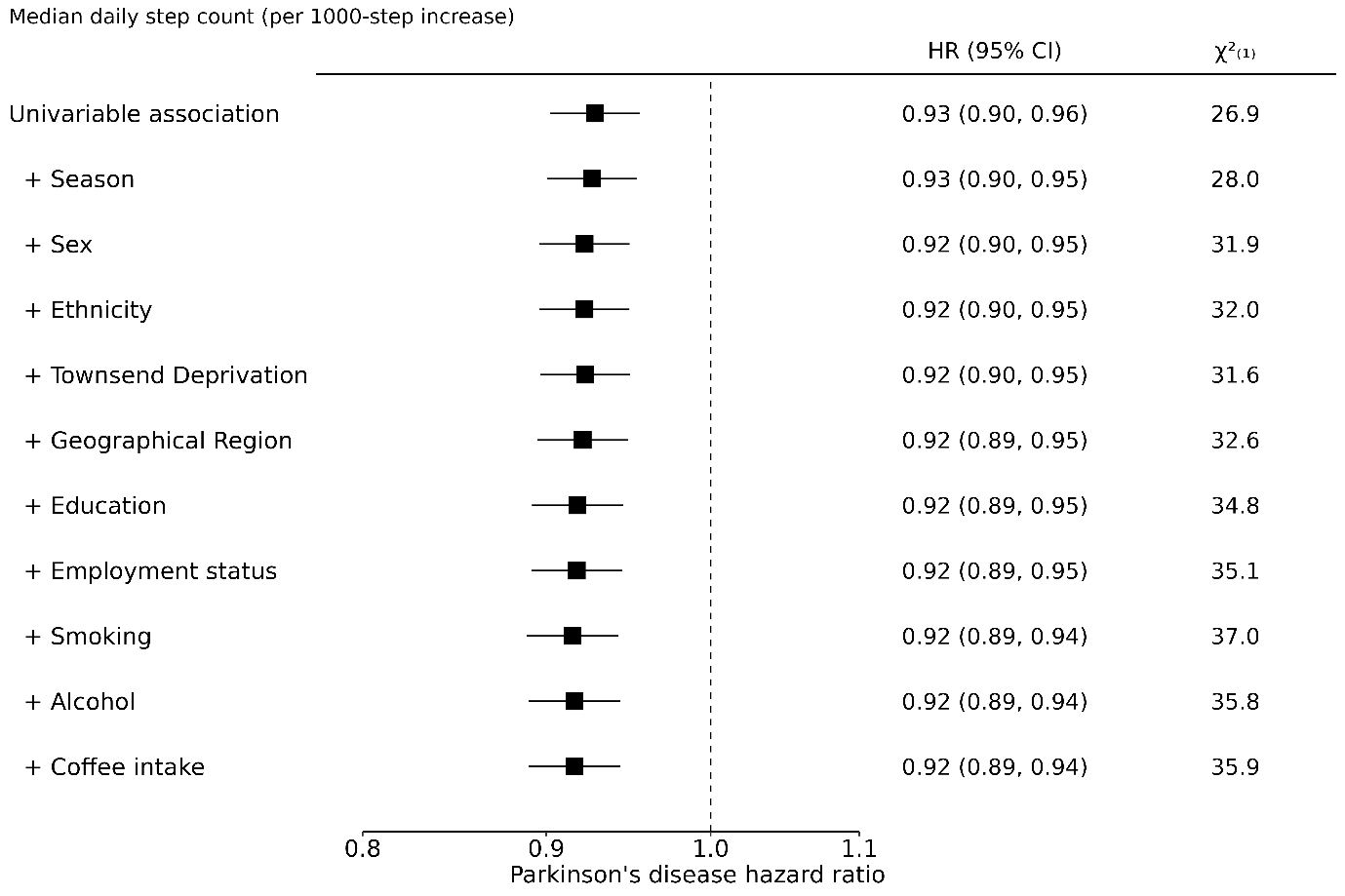


Supplementary Figure 3: The sequential adjustment of the association between 1,000 steps higher in imputed median daily step count and incident Parkinson's disease with age as timescale using all periods of follow-up. HR=Hazard ratio, CI=Confidence interval, $\chi_{(1)}^{2}$=chi squared using likelihood ratio test for one degree of freedom.


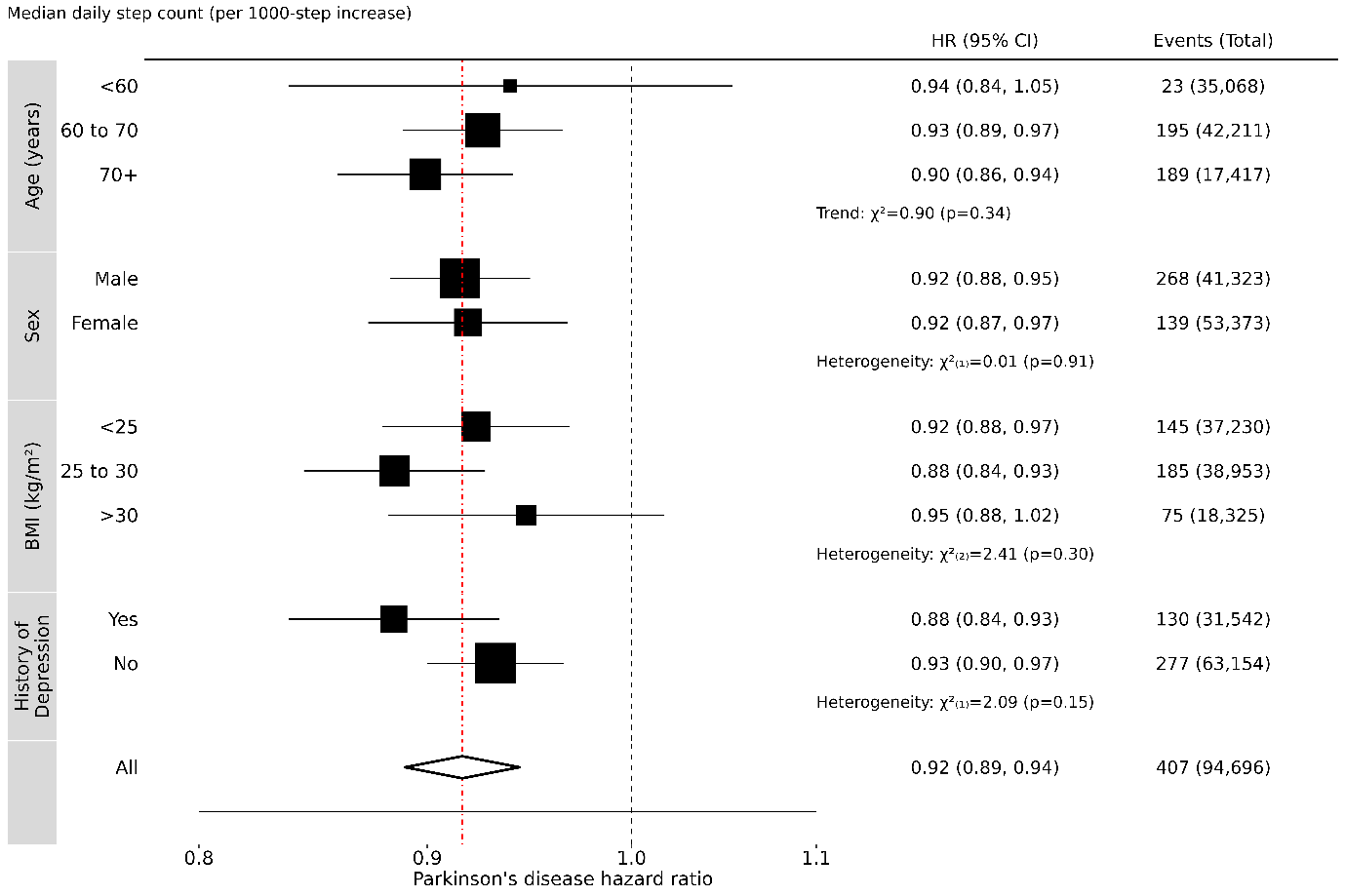


Supplementary Figure 4: The association between 1,000 steps higher in imputed median daily step count and incident Parkinson's disease with age as timescale using all periods of follow-up within various subpopulations. Models are adjusted for season, sex (except for sex-based sub-populations), ethnicity, Townsend deprivation, geographic region, education, employment status, smoking, alcohol and coffee intake. For each category, the area of the square is inversely proportional to the log HR, which also determines the 95\% CI.BMI=Body Mass Index, HR=Hazard ratio, CI=Confidence interval.


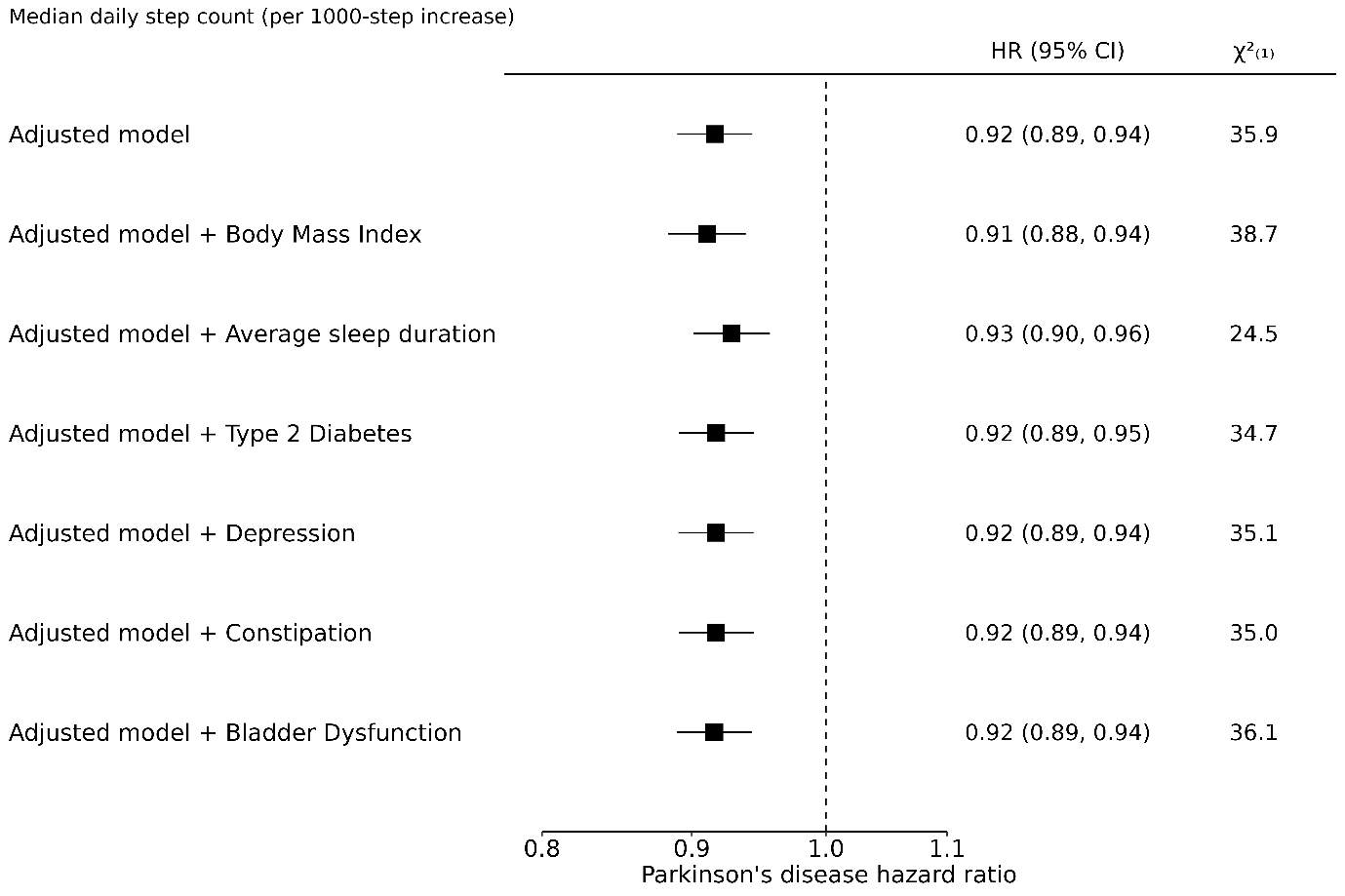


Supplementary Figure 5: The association between 1,000 steps higher in imputed median daily step count and incident Parkinson's disease with age as timescale using all periods of follow-up after adjustment for potential confounding factors. The adjusted model is adjusted for season, sex, ethnicity, Townsend deprivation, geographic region, education, employment status, smoking, alcohol and coffee intake. HR=Hazard ratio, CI=Confidence interval, $\chi_{(1)}^{2}$=chi squared using likelihood ratio test for one degree of freedom.


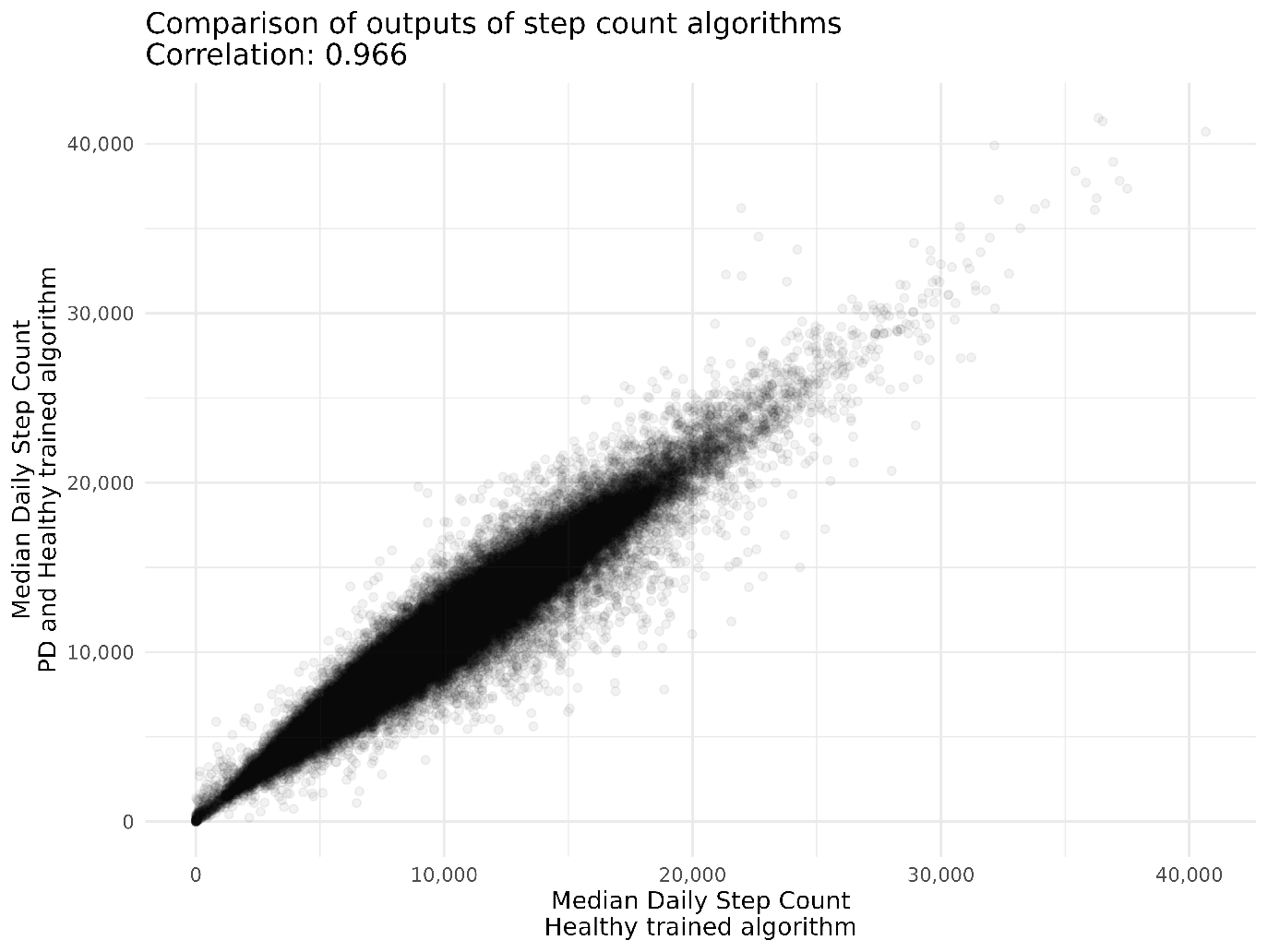


Supplementary Figure 6: Plot of the distributions of median daily step counts estimated on valid participants of the UK Biobank physical activity monitoring study. The x axis represents step counts produced by an algorithm trained only on the healthy, OxWalk study population. The y axis represents step counts produced by an algorithm trained on both the healthy, OxWalk study population, and the clinical PD, Michael J. Fox Foundation Levodopa Response study population.


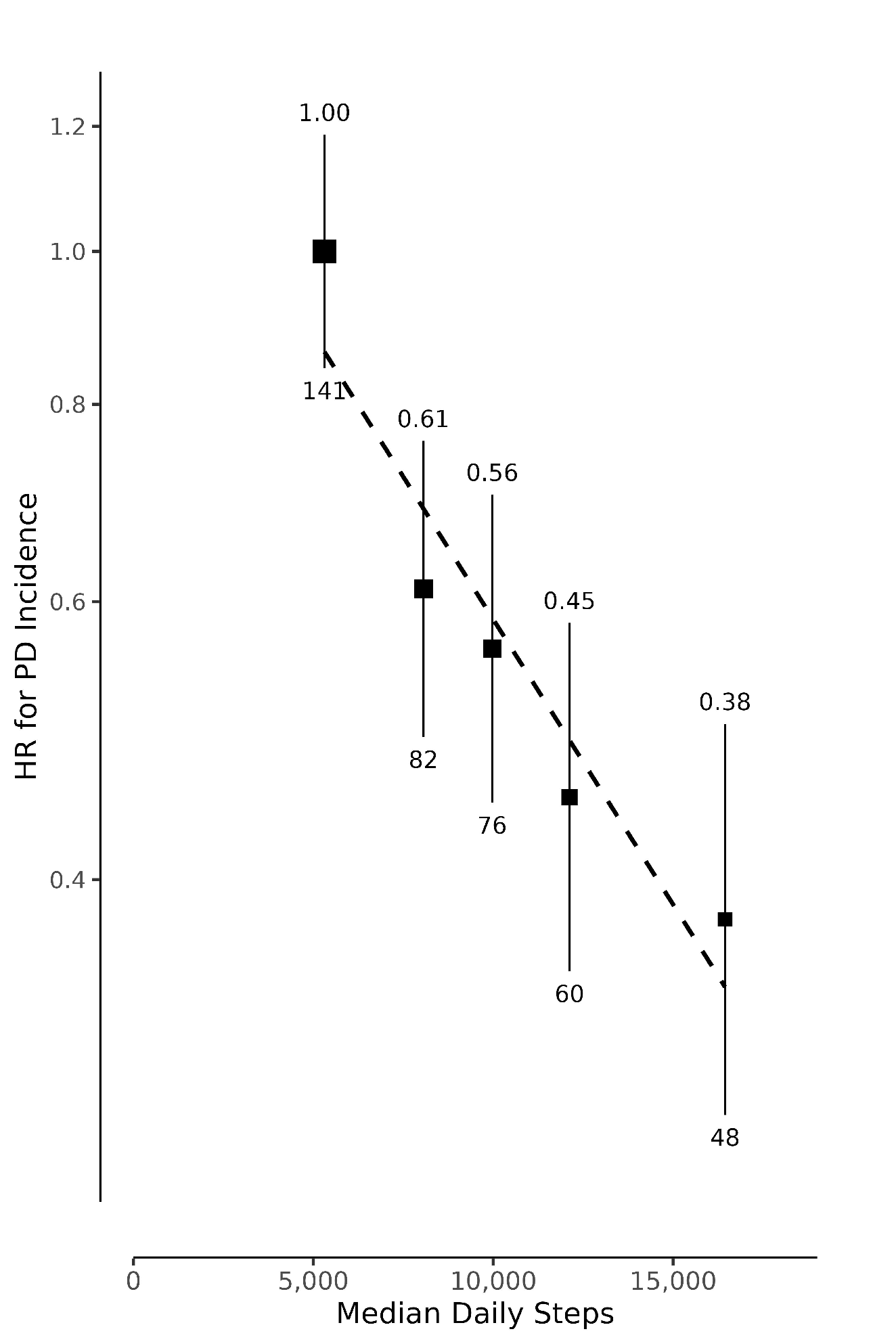


Supplementary Figure 7: Association between quintiles of median daily step count, derived using a **PD-trained algorithm,** and incident Parkinson’s disease. Hazard ratios (HR) and 95% group-specific confidence intervals (CI) were calculated using age as a timescale, adjusted for season of wear, sex, ethnicity, Townsend deprivation index, geographic region, education, employment, alcohol intake, smoking status and coffee intake. HR is above, and the number of events is plotted below each data point. The HR (95% CI) per 1,000 additional daily steps is 0.91 (0.89 – 0.94).


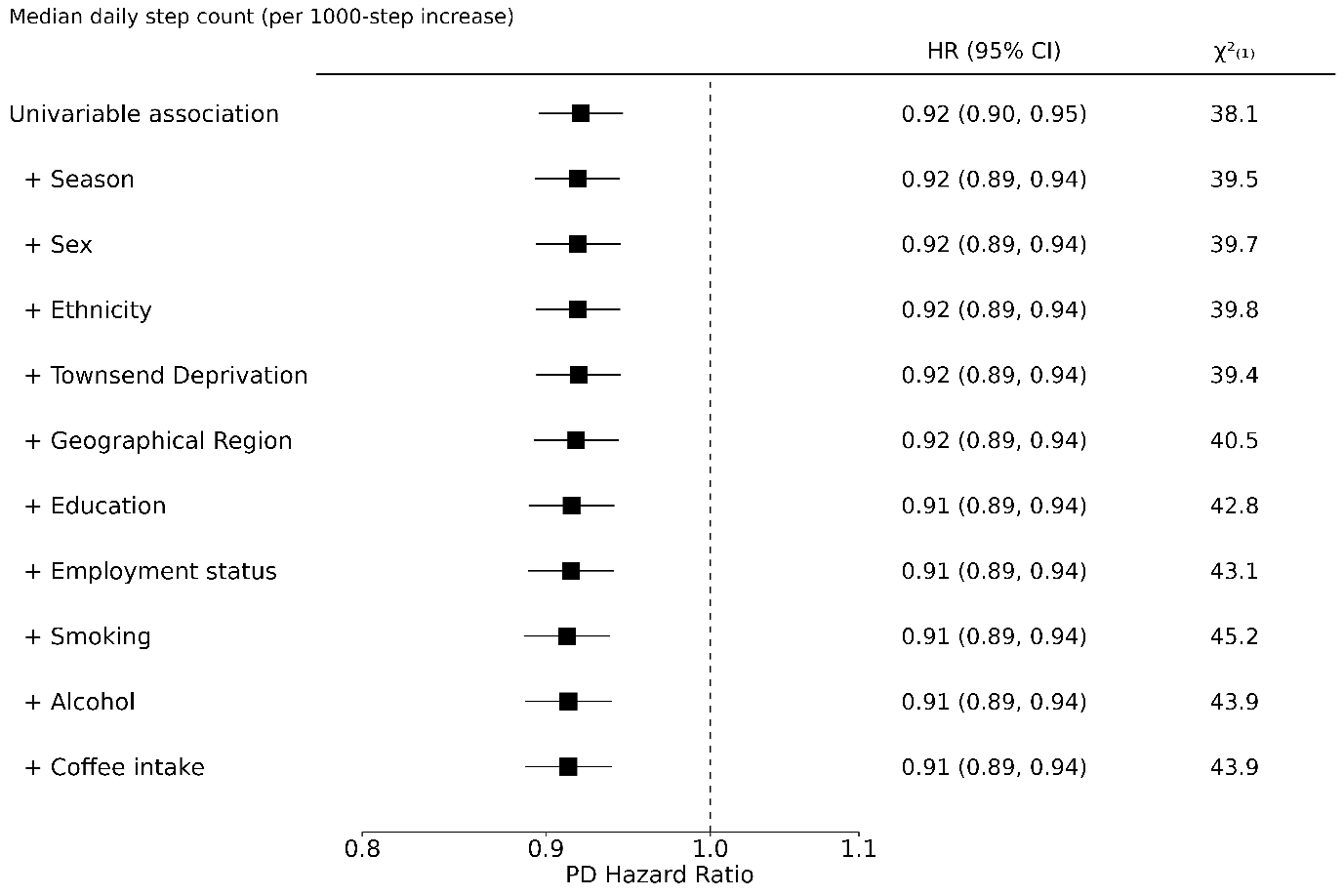


Supplementary Figure 8: The sequential adjustment of the association between 1,000 steps higher in imputed median daily step count, derived using a **PD-trained algorithm**, and incident Parkinson's disease with age as timescale using all periods of follow-up. HR=Hazard ratio, CI=Confidence interval, $\chi_{(1)}^{2}$=chi squared using likelihood ratio test for one degree of freedom.


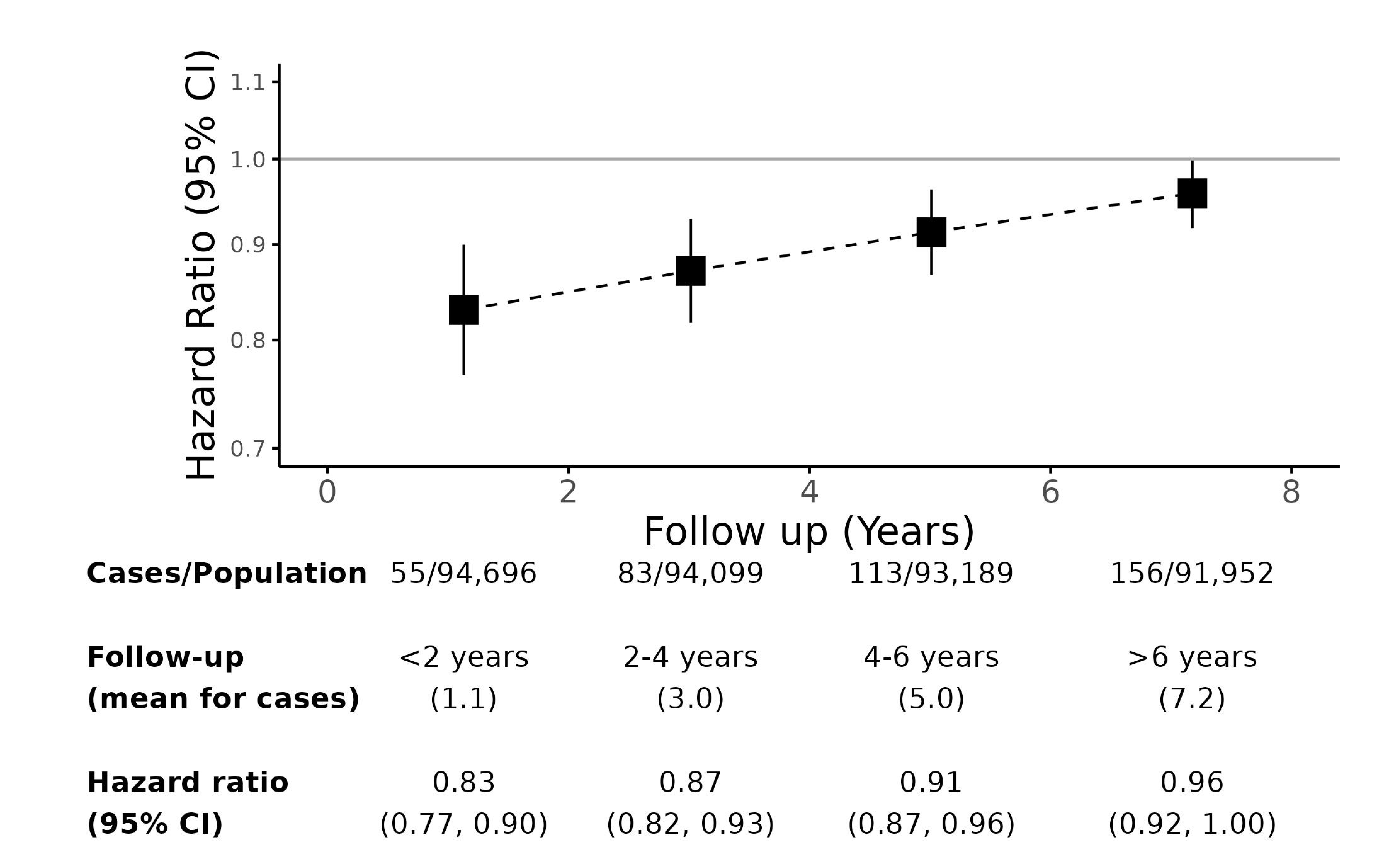


Supplementary Figure 9: Hazard ratio for incident Parkinson's disease by period of follow-up per 1,000 median daily steps higher, derived using a **PD-trained algorithm**. Models use age as timescale, and are adjusted for season of wear, sex, Townsend deprivation index, geographic region of assessment centre, educational attainment, employment, smoking, alcohol and coffee consumption. CI=confidence interval.

# **Power Calculations**

For the subset of the population with at least 6 years of follow-up, of which 156 incident PD cases were found, an expected hazard ratio of 0.942 per 1,000 steps higher in median daily steps would be required to achieve a power of 0.800 or greater.
